# Comparison of the Fracture Resistance and Fracture Patterns of Root-Filled Maxillary Premolars Restored with Different Post Systems and Composite-Based Post Space Restorations: An in Vitro Study

**DOI:** 10.34172/joddd.44391

**Published:** 2026-06-30

**Authors:** Sulhi Abidin, Hanna Nadhirah Mohd Radzi, Muhamad Sharifuddin Mat Daud, Norfaezah Ahmad, Izzuddin Ahmad Nadzirin, Galvin Sim Siang Lin

**Affiliations:** ^1^Department of Prosthodontics, Kulliyyah of Dentistry, International Islamic University Malaysia, Kuantan Campus, 25200, Pahang, Malaysia; ^2^Department of Restorative Dentistry, Kulliyyah of Dentistry, International Islamic University Malaysia, Kuantan Campus, 25200, Pahang, Malaysia; ^3^Department of Biomedical Science, Kulliyyah of Allied Health Sciences, International Islamic University Malaysia, Kuantan Campus, 25200, Pahang, Malaysia

**Keywords:** Endodontics, Fiber post, Metal post, Prosthodontics, Root canal, Tooth fracture

## Abstract

**Introduction:**

Root-filled teeth are structurally and biomechanically weakened, and the choice of post-endodontic restoration is important. This study compared the fracture resistance and fracture patterns of root-filled maxillary premolars restored with different post systems and composite-based post space restorations.

**Methods:**

One hundred extracted maxillary second premolars were endodontically treated and randomly allocated to five groups (n=20): metal post (group A), fiber post (group B), flowable composite resin in the post space (group C), fiber-reinforced composite resin in the post space (group D), and composite resin restoration without post with a glass ionomer orifice barrier (control, group E). All the specimens received a standardized mesio-occluso-distal (MOD) cavity preparation with preservation of the buccal wall and were restored with nanohybrid composite before axial loading in a universal testing machine to determine fracture resistance. Meanwhile, fracture patterns were classified under a stereomicroscope.

**Results:**

Mean (±standard deviation) fracture resistance values were metal post (896.26±335.55 N), fiber post (1046.64±336.65 N), flowable composite resin (937.94±323.83 N), fiber-reinforced composite resin (799.90±244.43 N), and control (973.54±357.33 N), with no statistically significant differences between the groups (*P*=0.175). Metal and fiber post groups predominantly exhibited favorable, restorable Class 2 fractures, whereas flowable composite resin, fiber-reinforced composite resin, and control groups mainly demonstrated Class 3 or Class 4 fractures.

**Conclusion:**

Root-filled teeth restored with or without posts exhibited similar fracture resistance. Fiber and metal posts, however, produced more favorable fracture patterns, suggesting that posts can influence failure mode even without increasing overall fracture strength.

## Graphical Abstract

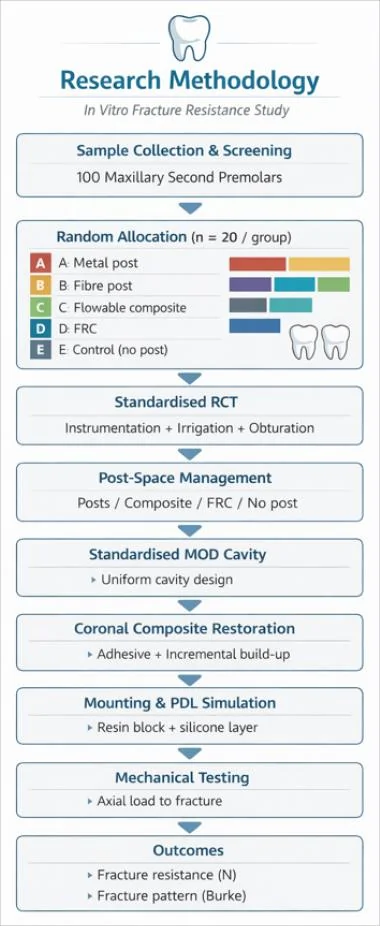

## Introduction

 Root canal treatment (RCT) involves removing the infected or severely inflamed pulp, cleaning and disinfecting the root canal system, and then filling and sealing the root canal space to prevent further infection. Root-filled teeth are structurally and biomechanically compromised due to recurrent endodontic disease, caries, prior restorations, access preparation, loss of marginal ridges and dehydration of dentin.^1^ These changes reduce the stiffness of the remaining tooth structure and alter its capacity to dissipate occlusal stresses, predisposing root-filled teeth to catastrophic fractures. Contemporary concepts for restoring root-filled teeth emphasize careful assessment of restorability, preservation of residual dentin, provision of a coronal seal, and adequate retention and resistance form for the definitive restoration.^2,3^

 Intraradicular posts have been used for many years, primarily to retain a core in teeth with extensive loss of coronal structure.^1,4^ Cast metal posts and prefabricated metal posts (e.g., stainless steel and titanium) demonstrate good retention and stiffness but have a modulus of elasticity that is far higher than that of dentin.^5^ This mismatch concentrates stresses at the post–dentin interface and has been associated with unfavorable, often irreparable root fractures.^6^ Earlier experimental studies also reported higher fracture resistance values for metal posts, but failure commonly occurred as unfavorable or below the crestal bone level, limiting options for retreatment or restoration.^7,8^

 The introduction of fiber-reinforced post systems has markedly changed restorative strategies.^9^ Glass, quartz, and carbon fiber posts exhibit an elastic modulus closer to that of dentin, promoting more homogeneous stress distribution and a higher incidence of favorable, restorable failures.^10^ A recent meta-analysis comparing fiber and metal posts concluded that, when a post is indicated, using a fiber post significantly reduces the risk of tooth fracture compared with no post, while providing survival outcomes comparable to those of metal posts.^11^ In parallel, there is growing interest in using fiber-reinforced composites (FRCs) and fiber ribbons in the coronal or radicular portion of the tooth as an alternative to, or adjunct to, conventional posts.^12^ A previous systematic review suggests that FRC generally improves the fracture resistance of root-filled teeth compared with conventional hybrid composite cores.^13^

 Despite significant advances in post materials and adhesive technologies, there remains no clear consensus on which post system offers the most favorable balance between fracture resistance and favorable failure patterns in root-filled teeth. With variations in tooth type, residual coronal structure, cavity design, post configuration, and restorative protocols contributing to inconsistent findings across studies.^1^ Moreover, although recent literature continues to introduce novel post materials and configurations,^11,14,15^ robust direct comparative data remain limited. Therefore, the present study addressed this gap by comparing the fracture resistance of root-filled maxillary premolar teeth restored with different post systems under controlled experimental conditions and by analyzing the mode of failure associated with each system. The null hypothesis of the present study was that neither the fracture resistance nor the fracture pattern of root-filled maxillary premolars would differ between those restored with metal posts, fiber posts, flowable composite resin in the post space, FRC in the post space, or composite resin restoration without posts.

## Methods

###  Sample Size Calculation

 The minimum required sample size was determined a priori using G*Power software version 3.1 (Heinrich Heine University, Düsseldorf, Germany), based on data from a previous study with comparable experimental conditions.^16^ An F-test (ANOVA: fixed-effects, one-way, omnibus) was selected as the statistical model. The calculated effect size (f) was 0.452, with the significance level (α) set at 0.05 and the desired power (1–β) set at 0.95. Under these parameters, the minimum total sample size was 100 teeth, corresponding to 20 samples per group for the five-group comparison.

###  Sample Collection and Selection

 A total of 100 extracted human maxillary second premolars (n = 20 per group) were collected for orthodontic or periodontal reasons from routine dental procedures performed at the Kulliyyah of Dentistry, IIUM. Following extraction, the teeth were cleaned using a scaler to remove calculus, soft tissue remnants, and debris. Each tooth was examined under an operating microscope (Leica M320, Leica Microsystems, Wetzlar, Germany) and radiographically screened to ensure compliance with the inclusion criteria. Only teeth with intact roots, a single canal, and no evidence of cracks, caries, restorations, calcifications, previous endodontic treatment, or root resorption were included. Tooth dimensions were verified using a digital Vernier caliper (Mitutoyo Corporation, Kanagawa, Japan) to match standard measurements for maxillary second premolars: buccolingual width (9.45 ± 0.20 mm), mesiodistal width (7.42 ± 0.20 mm), and occlusogingival height (9.30 ± 0.20 mm). All the selected samples were stored in 0.9% normal saline at room temperature until use.^17^

###  Group Allocation

 The teeth were randomly divided into five groups: four experimental and one control (n = 20 per group); group A: RCT + metal post + composite; group B: RCT + fiber post + composite; group C: RCT + flowable composite resin placed into post space; group D: RCT + FRC in post space; and group E (control): RCT + composite resin restoration (no post).

###  Root Canal Treatment (RCT)

 The RCT was conducted following a standardized protocol by a single operator. Access cavities were prepared using diamond burs (Shofu Inc., Kyoto, Japan) under water coolant. Root canal shaping was performed using the ProTaper Universal rotary system (Dentsply Sirona, Ballaigues, Switzerland) up to size X2 (#25, taper.06). Irrigation throughout instrumentation consisted of 5.25% sodium hypochlorite (NaOCl, Malay-Sino Chemical Industries, Johor, Malaysia). Final irrigation included 17% EDTA (Promega Corporation, Madison, WI, USA), followed by a final rinse of NaOCl. The root canals were dried using sterile paper points (Dentsply Maillefer, Ballaigues, Switzerland) and obturated using the single-cone technique with X2 matched-taper gutta-percha and AH Plus sealer (Dentsply DeTrey GmbH, Konstanz, Germany). Excess gutta-percha was cut and compacted to 2 mm apical to the cementoenamel junction (CEJ) using a heated plugger.

###  Post Space Preparation and Post Placement

 For groups A and B, the post space was prepared using the ParaPost drill system (Coltene/Whaledent Inc., Cuyahoga Falls, OH, USA) with a 1.5-mm drill to a depth of 12 mm. Metal posts (Group A) and fiber posts (group B) were cemented using a dual-cured resin cement (RelyX Ultimate, 3M ESPE, St. Paul, MN, USA) following manufacturer guidelines. In groups C and D, coronal gutta-percha was removed to a depth of 4 mm below the root canal orifice. Group C post spaces were restored with flowable composite resin (G-aenial Universal Flo, GC Corporation, Tokyo, Japan), while group D post spaces were restored using FRC (EverX Posterior, GC Corporation, Tokyo, Japan).

###  Standardized Coronal Cavity Preparation

 A uniform MOD-type cavity was prepared for all the samples by removing the mesial, distal, and palatal walls while preserving the buccal wall. Preparations were performed with a high-speed handpiece and tapered diamond bur under continuous water irrigation. A dental surveyor was used to ensure uniform orientation and reproducibility of cavity dimensions.

###  Coronal Restoration

 In group E, coronal gutta-percha was reduced to 2 mm below the CEJ before placing a glass ionomer cement as an orifice barrier (Fuji IX GP EXTRA, GC Corporation, Tokyo, Japan). For all teeth, cavity surfaces were etched with 37% phosphoric acid for 20 seconds, rinsed, and gently dried. A universal adhesive (Scotchbond Universal Adhesive, 3M ESPE) was applied and light-cured using Elipar^TM^ DeepCure-S (3M ESPE, USA) for 20 seconds. The cavities were restored incrementally with A2B nanohybrid composite resin (GC Corporation, Japan), curing each increment for 20 seconds. Finishing and polishing were performed using the Enhance Finishing and Polishing System (Dentsply Caulk, Milford, DE, USA). The restored teeth were stored in normal saline until mounting.

###  Sample Mounting and Periodontal Ligament Simulation

 Each tooth was coated with petroleum jelly and mounted in polyurethane resin (AlphaDie MF, Schütz Dental GmbH, Rosbach, Germany) within copper cylinders, embedding the root up to 2 mm apical to the CEJ. Once the resin set, the teeth were removed, and the socket was lined with light-body silicone (President Plus®, Coltene Holding AG, Altstätten, Switzerland) to simulate the periodontal ligament.^16^ The teeth were reinserted until the silicone set. Excess material was trimmed, and only specimens with mobility grade 0 were included.

###  Mechanical Testing

 Fracture resistance testing was performed using a universal testing machine (Instron 3366, Instron Corp., Norwood, MA, USA). Each mounted tooth was positioned so that its long axis was aligned parallel to the loading crosshead.^10^ A 4.24-mm diameter stainless steel ball applied an axial compressive load at a crosshead speed of 1 mm/min. A 1-mm dental dam sheet was placed between the loading ball and the occlusal surface to distribute occlusal stresses evenly. The load was applied until catastrophic fractures occurred, and the maximum force at failure (N) was recorded. All the fractured samples were examined under a stereomicroscope to evaluate the fracture pattern. Fracture patterns were classified according to Burke’s classification (Types 1–4) and further differentiated between favorable (above CEJ) and unfavorable (below CEJ) outcomes.^18^

###  Statistical Analysis

 Statistical analysis was performed using SPSS 19.0 (IBM Corp., Chicago, USA). Mean fracture resistance and standard deviation were calculated for each group. One-way ANOVA was used to assess differences between the groups, followed by post hoc Tukey tests for pairwise comparisons. Statistical significance was set at *P* = 0.05.

## Results

 The mean fracture resistance values for the metal post (group A), fiber post (group B), flowable composite resin (group C), FRC (group D), and control groups (group E) were 896.26 ± 335.55 N, 1046.64 ± 336.65 N, 937.94 ± 323.83 N, 799.90 ± 244.43 N, and 973.54 ± 357.33 N, respectively (Figure 1). One-way ANOVA showed no statistically significant differences between the five groups (*P* = 0.175). Overall, root-filled premolars restored with metal and fiber posts predominantly exhibited Class 2 fractures, while flowable composite specimens most frequently demonstrated Class 4 fractures (Figure 2). FRC and control groups mainly showed Class 3 fractures, with coronal fractures extending into the periodontal region. Moreover, metal and fiber post groups showed predominantly favorable failures, while flowable composite resin, FRC, and control groups exhibited mainly unfavorable or non-restorable failures (Figure 3).

**Figure 1:**
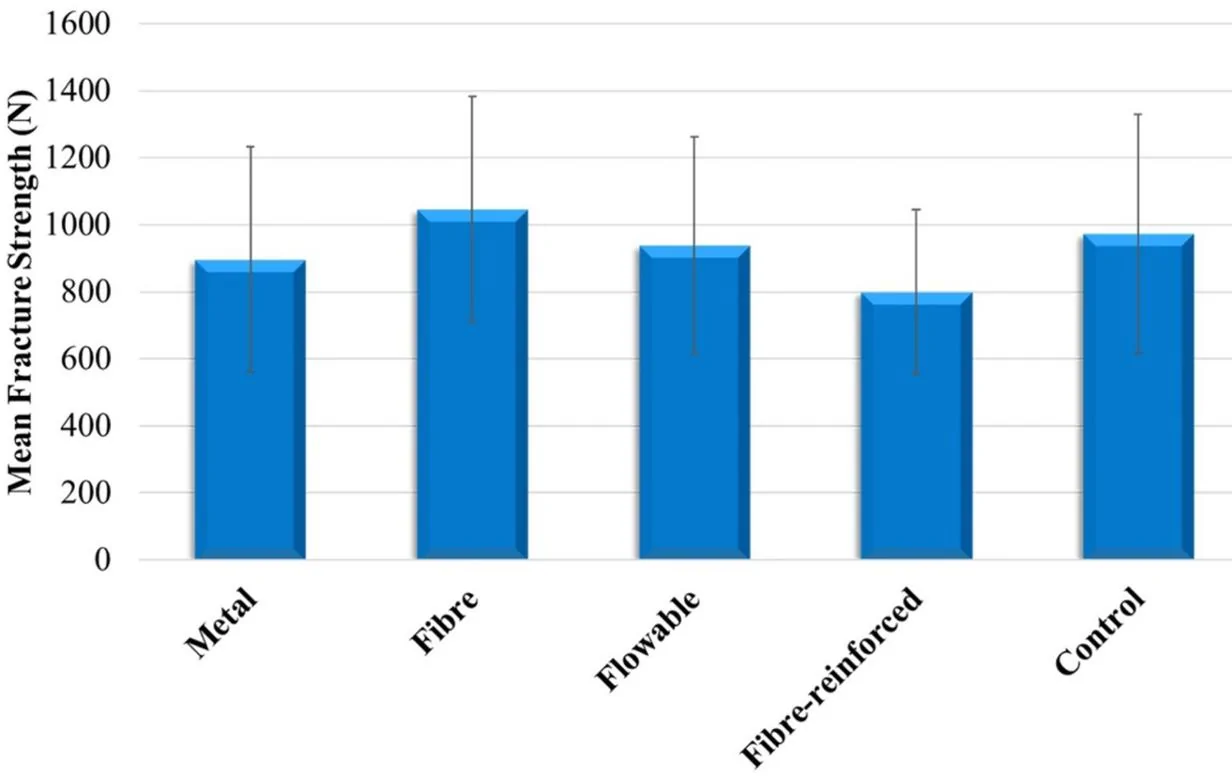
Mean fracture resistance of teeth restored with various post systems and post space alternatives

**Figure 2:**
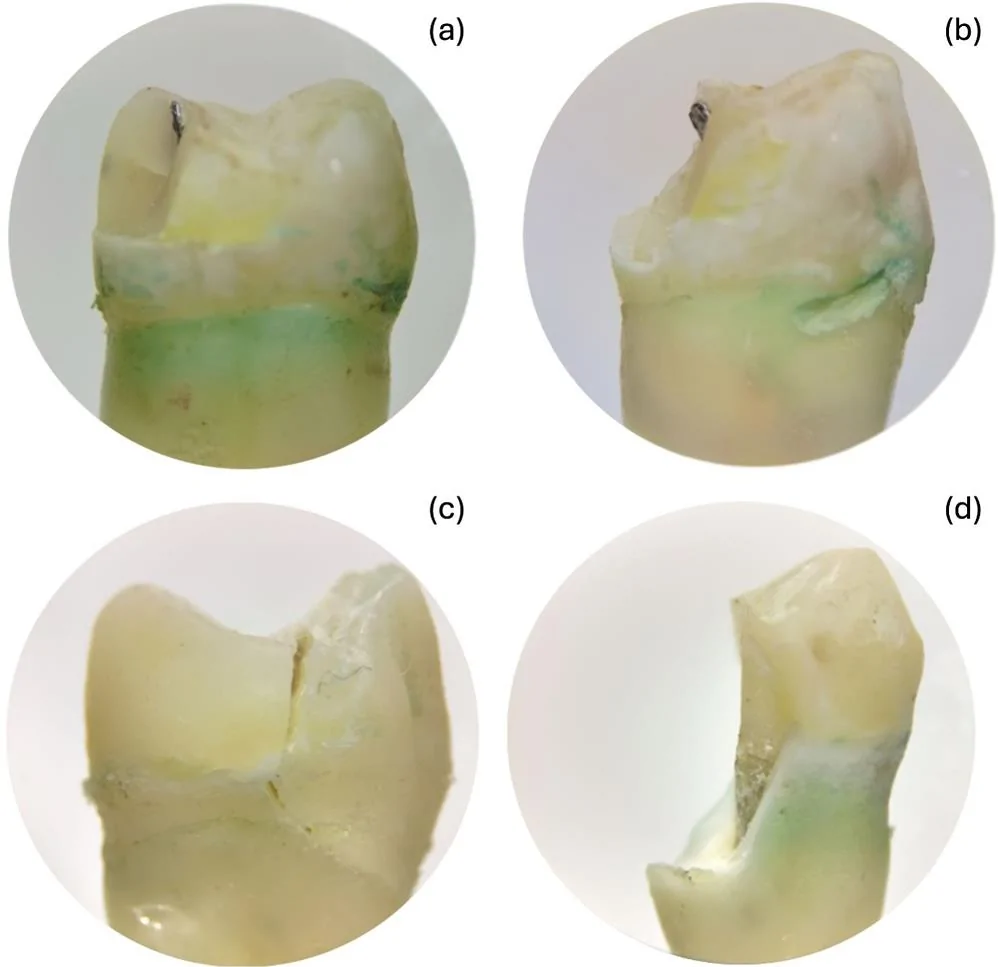
Fracture patterns according to Burke’s classification: (a) Class 1: minimal coronal fracture; (b) Class 2: coronal fracture with cohesive failure; (c) Class 3: coronal fracture extending into the periodontal region; (d) Class 4: combined coronal and vertical root fracture

**Figure 3:**
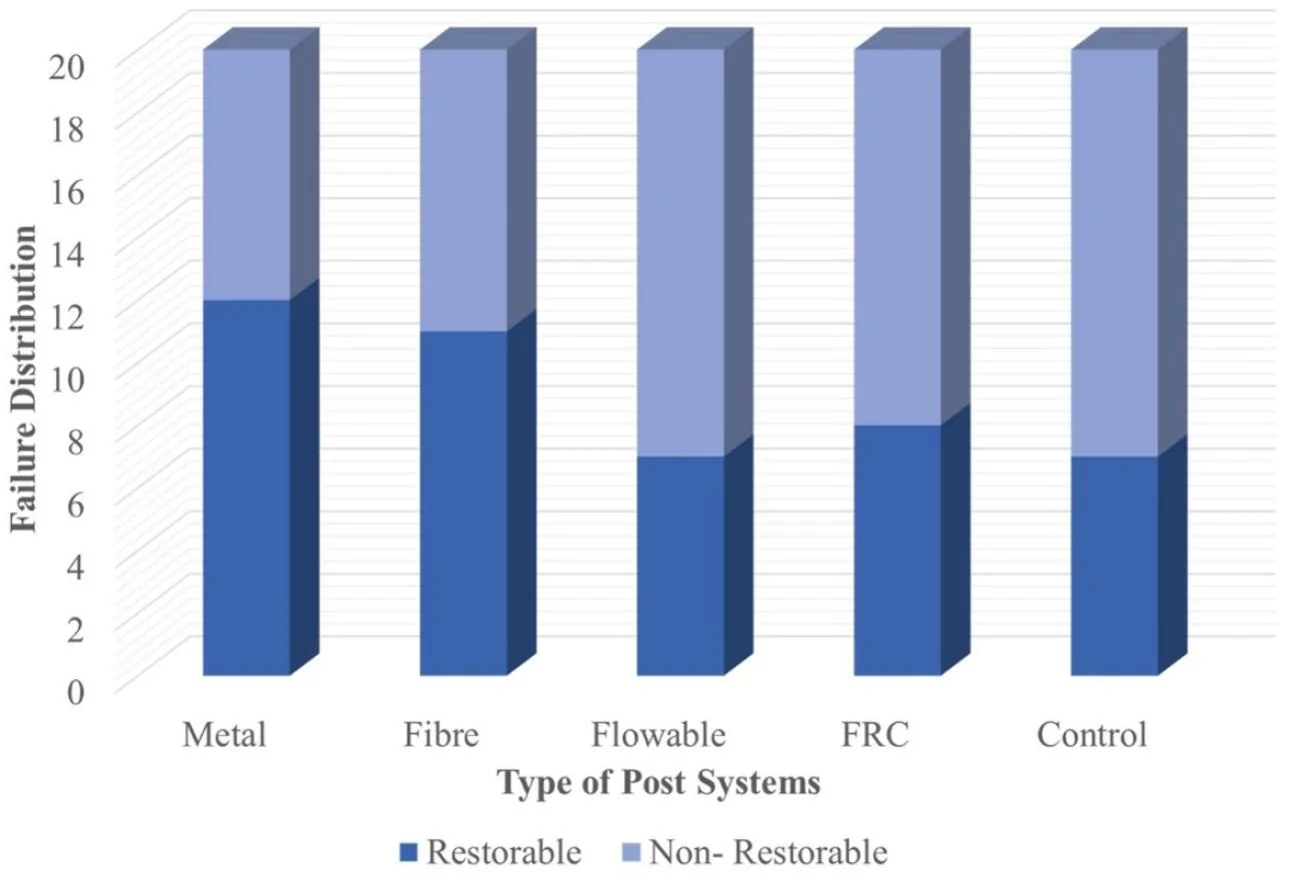
Failure distribution among post systems and post space restorative alternatives

## Discussion

 The present study evaluated the fracture resistance and fracture patterns of maxillary premolars restored with four different post systems. Fracture resistance reflects a tooth’s ability to resist crack initiation and propagation under functional loading,^16,19^ and it is a critical determinant of the longevity of root-filled teeth. Root-filled teeth are mechanically compromised due to loss of structural integrity from caries, access preparation, root canal shaping, and reduced dentinal moisture content.^1,20^ This reduced toughness makes maxillary premolars, already biomechanically vulnerable due to their cusp anatomy and involvement in lateral guidance, especially prone to fracture under masticatory forces.^21^ Posts are traditionally placed not to strengthen roots, but to retain the core and assist in distributing functional stresses, potentially reducing cervical stress concentrations.^22^

 Interestingly, no statistically significant differences were found between the groups, and thus, the null hypothesis was accepted. This outcome is consistent with previous reports showing that when a portion of coronal tooth structure is preserved, the choice of post system has minimal impact on the overall fracture resistance of root-filled teeth.^15,23^ Supporting this, recent finite element analysis demonstrates that increasing ferrule height markedly reduces stress concentrations, particularly with rigid post materials.^23^ Similarly, a recent laboratory study on root-filled mandibular premolars showed that increasing ferrule thickness from 0 to 1.5 mm significantly enhanced fracture resistance after thermomechanical fatigue.^15^ These highlight that residual tooth structure and the ferrule effect often play a more dominant biomechanical role than the post material itself. Furthermore, in line with these findings, the recent European Society of Endodontology position statement on the restoration of root-filled teeth emphasizes that long-term survival is governed primarily by the amount of residual tooth structure and an adequate ferrule, with posts serving mainly for core retention.^3^

 Beyond the established influence of ferrule presence highlighted earlier, the inherent structural configuration of the specimens in the present study may also help clarify why fracture resistance did not differ significantly between post systems. All specimens in the present study retained a single coronal wall (buccal wall) after MOD cavity preparation. Although not a formal ferrule, this surviving wall provides a ferrule-like effect, acting as a structural bracing element that reduces cuspal deflection and redistributes tensile stresses.^24^ Importantly, a recent meta-analysis of 17 in vitro studies demonstrated that while complete ferrules produce the highest fracture resistance, partial ferrules, particularly 2 mm buccal or lingual walls, offer resistance comparable to complete ferrules and significantly outperform teeth with no ferrule at all.^25^ It is also important to recognize that dentin itself is not biomechanically uniform; variations in dentinal hardness and microstructure have been shown to influence crack initiation and propagation under mechanical loading, which may further moderate how stresses are dissipated within the tooth–restoration complex.^26^ The buccal wall in the present study, therefore, likely acted as a stabilizing factor that mitigated differences between post systems.

 Remaining coronal dentin also strengthens the tooth–restoration complex by providing more surface area for bonding and improving the rigidity of the composite core. In line with this, an ex vivo study reported that fracture resistance increased significantly when ≥ 2 mm of buccal and lingual coronal dentin was preserved and that, once this dentin was retained, teeth restored with various posts exhibited broadly similar fracture resistance.^27^ When such structural support is present, the post assumes a predominantly retentive rather than reinforcing role, which helps to explain the comparable fracture resistance observed across all groups in the present study. Despite the lack of statistical significance, fiber posts exhibited the highest mean fracture resistance. This may be attributed to their elastic modulus resembling that of dentin, which enables more homogeneous deformation and efficient stress dissipation along the root.^10,28^ Fiber posts also possess high flexural strength and bond effectively with resin cement and composite cores, creating a cohesive monoblock that may resist crack propagation more effectively than other post types.^9^

 Fracture pattern analysis provides additional clinically relevant insights. Teeth restored with fiber and metal posts in the present study exhibited mainly favorable fracture patterns (Class 2). The presence of an intra-radicular post may help absorb and redirect occlusal forces coronally, reducing the likelihood of deep root fractures.^29^ Such a finding corroborates a systematic review and meta-analysis showing that fiber posts not only increase the fracture resistance of root-filled maxillary premolars but also shift failures toward more restorable fracture patterns compared with teeth restored without posts.^30^ Conversely, flowable composite resin, FRC, and control specimens showed mostly non-restorable fractures (Class 3 and 4). This can be explained by the composite mechanics literature, where impact behavior and failure mode are governed not only by the intrinsic toughness of the fiber-reinforced material but also by fiber content, orientation, and structural configuration.^31^ Nevertheless, in complex tooth‒restoration assemblies, these factors may override the crack-arresting potential of flowable FRC, predisposing them to catastrophic root-level fractures despite their favorable material properties.

 The present study is not without limitations. First, the present in vitro study did not fully replicate the oral environment. Thermocycling, cyclic loading, moisture variability, long-term bonding degradation, and complex occlusal dynamics were not incorporated. Only maxillary second premolars with a single retained wall were tested, limiting the generalizability to situations with different cavity designs or varying amounts of residual tooth structure. The study also did not evaluate the effects of full crowns, varying ferrule heights, or different post diameters. Future research should incorporate thermomechanical aging, evaluate teeth with different remaining wall configurations, and explore newer adhesive strategies and post materials. Clinical trials and finite element analyses would further clarify stress distribution patterns and long-term behavior of post-restored teeth.

## Conclusion

 In conclusion, the present study showed that when a buccal wall is preserved, root-filled maxillary premolars restored with different post systems or without posts exhibit similar fracture resistance, confirming that residual coronal dentin and a ferrule-like effect are more influential than post type. However, fiber and metal posts produced more favorable, restorable fracture patterns, indicating that posts can beneficially influence failure mode even without increasing overall fracture strength. Clinically, these findings reinforce that preserving tooth structure and achieving a ferrule remain paramount, with posts serving mainly for core retention. Further studies incorporating aging and varied cavity designs are recommended to enhance clinical applicability.

## Competing Interests

 The authors declare no conflicts of interest.

## Data Availability Statement

 The datasets are not publicly available but can be obtained from the corresponding author upon reasonable request.

## Declaration of Generative AI and AI-assisted Technologies

 During the preparation of this work, the authors used Grammarly to assist with English grammar, spelling, punctuation, and language clarity, and QuillBot to support sentence rephrasing for improved readability and fluency. These tools were used solely for language refinement. After using these tools, the authors carefully reviewed, revised, and edited the manuscript as needed and took full responsibility for the accuracy, integrity, and content of the published article.

## Ethical Approval

 Ethical approval for this study was obtained from the Research Ethics Committee of the International Islamic University Malaysia (IIUM), approval code IIUM/504/14/11/2/IREC 2019-214.
